# Supplementary material for: A Retrospective, Monocentric Study Comparing Co and Secondary Infections in Critically Ill COVID-19 and Influenza Patients
Source: Antibiotics (Basel). 2022 May 24;11(6):704. doi: 10.3390/antibiotics11060704 (PMC9219538; doi:10.3390/antibiotics11060704)
Supplement: Supplementary file 1 [file antibiotics-11-00704-s001.zip › antibiotics-1716636-SI.pdf]

SUPPLEMENTARY DATA

Table S1. Patients characteristics according to their co-infection status.

	COVID-19 n=57		Influenza n=55	
	**Co-infected (n=13)**	**Non co-infected (n=44)**	**Co-infected (n=40)**	**Non co-infected (n=15)**
Sex , n (%)	8 (62)	33 (75)	22 (55)	8 (53)
Age (years)	63 [53-67]	61 [53-71]	64 [52-73]	77 [66-82]
Current smokers, n(%)	2 (15)	4 (9)	19 (48)	4 (27)
Chronic pulmonary disease, n(%)	4 (30)	13 (30)	19 (48)	8 (53)
Obesity (BMI ≥ 30 kg/m2), n(%)	5 (38)	14 (32)	9 (23)	5 (33)
Arterial hypertension, n(%)	7 (54)	30 (68)	22 (55)	12 (80)
Diabetes, n(%)	0 (0)	11 (25)	9 (23)	7 (47)
Charlson Comorbidity index	4 [3-5]	3 [1-5]	4 [3-5]	6 [4-7]
Immunosuppressive therapy, n(%)	4 (30)	2 (5)	17 (43)	7 (47)
Solid organ transplant, n(%)	1 (8)	2 (5)	5 (13)	1 (7)
Baseline SOFA	8 [6-12]	7 [3-9]	6 [3-10]	5 [4-9]
Baseline SAPS 3	54 [48-68]	56 [45-67]	62 [54-67]	61 [48-73]
Baseline PaO_2/FiO_2 ratio	140 [103-193]	140 [101-191]	148 [110-190]	176 [117-228]

Continuous variables are reported as median [Interquartile range] and categorical variables,as numbers (percentages).

Table S2. Patients characteristics according to their secondary infection status.

	COVID-19 n=57		Influenza n=55	
	**Secondary infection (n=37)**	**No secondary infection (n=20)**	**Secondary infection (n=16)**	**No secondary infection (n=39)**
Sex , n (%)	29 (78)	12 (60)	9 (56)	21 (54)
Age (years)	59 [53-65]	67 [53-75]	62 [53-68]	70 [55-81]
Current smokers, n(%)	4 (11)	2 (10)	7 (44)	16 (41)
Chronic pulmonary disease, n(%)	11 (30)	6 (30)	6 (38)	21 (54)
Obesity (BMI ≥ 30 kg/m2), n(%)	17 (46)	3 (15)	4 (25)	11 (28)
Arterial hypertension, n(%)	25 (68)	12 (60)	9 (56)	25 (64)
Diabetes, n(%)	8 (22)	3 (15)	3 (19)	13 (33)
Charlson Comorbidity index	3 [1-5]	5 [3-5]	3 [2-5]	5 [3-6]
Immunosuppressive therapy, n(%)	5 (14)	1 (5)	6 (38)	18 (46)
Solid organ transplant, n(%)	3 (8)	0 (0)	1 (6)	5 (13)
Baseline SOFA	8 [5-10]	3 [2-6]	6 [2-10]	6 [4-10]
Baseline SAPS 3	55 [46-66]	56 [46-68]	64 [56-76]	61 [50-69]
Baseline PaO_2/FiO_2 ratio	120 [88-168]	190 [134-276]1	148 [134-158]	157 [109-224]
Vasopressors, n(%)	34 (92)	0 ()	13 (81)	25 (64)
ECMO, n(%)	13 (35)	7 ()	7 (44)	1 (3)

Continuous variables are reported as median [Interquartile range] and categorical variables,as numbers (percentages).
